# Association between NLPR1, NLPR3, and P2X7R Gene Polymorphisms with Partial Seizures

**DOI:** 10.1155/2017/9547902

**Published:** 2017-04-19

**Authors:** Haidong Wang, Pengfei Xu, Dehua Liao, Ruili Dang, Xin He, Yujin Guo, Pei Jiang

**Affiliations:** ^1^Department of Pharmacy, The First People's Hospital of Lianyungang, The Affiliated Hospital of Kangda College of Nanjing Medical University, Jiangsu, Lianyungang 222002, China; ^2^Institute of Clinical Pharmacy & Pharmacology, Jining First People's Hospital, Jining Medical University, Jining 272000, China; ^3^Department of Pharmacy, Hunan Cancer Hospital, Central South University, Changsha 410011, China; ^4^Department of Pharmacy, Second Xiangya Hospital, Central South University, Changsha 410010, China

## Abstract

*Objectives*. Clinical and experimental evidence has clarified that the inflammatory processes within the brain play a pivotal role in the pathophysiology of seizures and epilepsy. Inflammasomes and P2X7 purinergic receptor (P2X7R) are important mediators during the inflammatory process. Therefore, we investigated the possible association between partial seizures and inflammasomes NLPR1, NLRP3, and P2X7R gene polymorphisms in the present study.* Method*. A total of 163 patients and 201 health controls were enrolled in this study and polymorphisms of NLPR1, NLRP3, and P2X7R genes were detected using polymerase chain reaction- (PCR-) ligase detection reaction method.* Result*. The frequency of rs878329 (G>C) genotype with C (CG + CC) was significantly lower among patients with partial seizures relative to controls (OR = 2.033, 95% CI = 1.290–3.204, *p* = 0.002 for GC + CC versus GG). Intriguingly, we found that the significant difference of rs878329 (G>C) genotype and allele frequency only existed among males (OR = 2.542, 95% CI = 1.344–4.810, *p* = 0.004 for GC + CC versus GG), while there was no statistically significant difference among females. However, no significant results were presented for the genotype distributions of rs8079034, rs4612666, rs10754558, rs2027432, rs3751143, and rs208294 polymorphisms between patients and controls.* Conclusion*. Our study demonstrated the potentially significant role of NLRP1 rs878329 (G>C) in developing susceptibility to the partial seizures in a Chinese Han population.

## 1. Introduction

Epilepsy is an electrical disturbance in the brain characterized by uncontrollable, excessive or synchronous neuronal activity and an enduring predisposition to produce seizures [1, 2]. It always accompanies the emotional and cognitive dysfunction and affects about 70 million people worldwide, accounting for approximately 1% of the whole population at present [3]. Partial seizure (also called focal seizure) is one of the most common forms of epilepsy and is initially focused in just one part of the brain. There are two types of partial seizures: simple partial seizures and complex partial seizures. The symptoms of the partial seizures depend on the base of where the seizure occurs [4, 5].

Over the last decade, accumulating clinical and experimental evidence has clarified that the inflammatory processes within the brain play a pivotal role in the pathophysiology of seizures and epilepsy [6–8]. Among all the proinflammatory cytokines, the release of interleukin- (IL-) 1*β* is considered as central to the initiation and regulation of inflammation [9]. Inflammasomes, multiprotein complexes consisting of proinflammatory caspase, nucleotide-binding oligomerization domain-like receptor family members containing pyrin domain (NLRP), and the adaptor molecule apoptosis-associated speck-like protein, have proved their vital role in the process of the generation of the IL-1*β* [10, 11]. NLRP1 and NLRP3 are the two best characterized members of inflammasomes and their activation during epilepsy has been documented in both basic and clinical researches [12–15]. The exact process of the activation of NLRP1 and NLRP3 is unclear, but one potential mechanism is the reduction of potassium ions in cells resulting from the dilation of the P2X7 purinergic receptor- (P2X7R-) gated channel [16, 17]. In addition, it has been indicated that P2X7R plays a pivotal role in the pathophysiology of several neuropsychiatric disorders, including the seizures [18, 19].

Therefore, the aim of this present study was to investigate the role of NLRP1 (rs8079034, rs878329), NLRP3 (rs4612666, rs10754558, and rs2027432), and P2X7R (rs3751143, rs208294) gene polymorphisms in Chinese partial seizures population.

## 2. Materials and Methods

### 2.1. Subjects

The study cohort consisted of 163 unrelated Chinese patients (male : female = 89 : 74) younger than 19 years and they were enrolled at the outpatient clinic of the Second Xiangya Hospital of Hunan Province from November 2013 to December 2015. The patients were diagnosed using the “Classification of Epilepsies and Epileptic Syndromes” proposed by the Commission on Classification and Terminology of the International League Against Epilepsy. The mean age at seizure onset was 4.7 ± 2.9 years (range 0.08–12.9 years), and the mean duration of epilepsy was 3.1 ± 2.3 years (range 0–6.6 years). The epileptologists from Second Xiangya Hospital made the diagnosis of partial seizures on the basis of a range of clinical seizure semiology, interictal and ictal EEG, typical mesial temporal auras, and MRI criteria for each patient. There were no mass malformations of cortical development, cerebral tumor, or traumatic brain lesion or injury from vascular malformation for any patients. Anyone who had mental retardation, psychiatric difficulties, or obvious mental retardations was ruled out.

201 healthy individuals (males : female = 99 : 102), 14 to 48 years of age, ethnicity and sex matched from the same area, were recruited to participate in the study as a control group. None of the control participants had any history of central nervous system diseases or any other medical disorders. The study protocol was approved by the local institutional ethics committee, and written informed consent was obtained from each participant in this study.

### 2.2. DNA Isolation and Genotyping

Genomic DNA was isolated from about 1 ml EDTA anticoagulated venous blood samples by SQ Blood DNA Kit II (D0714-250, Omega Bio-Tek, Norcross, GA) according to the manufacturer's instructions. The polymerase chain reaction- (PCR-) ligase detection reaction method was employed to genotype all DNA samples. The PCR of the 7 target single-nucleotide polymorphisms was amplified by the primers shown in Table 1. A DNA sequencer was applied to detect the amplified products. No less than 10% of the samples were randomly picked out and retested to verify the validity of this procedure, and the results of the retested samples were consistent with those obtained from the original sample.

### 2.3. Statistical Analysis

All genotyping results in partial seizures patients and controls were tested for Hardy–Weinberg Equilibrium (HWE) by applying Chi-square test (*χ*^2^ test). Chi-square statistics (*χ*^2^ test) were used to compare the statistical differences in genotype distributions and allele frequencies between patients and controls. The associations between the genotypes and the partial seizures were evaluated via the odds ratio (OR), with a 95% confidence interval (CI) (95% CI), and a two-tailed *p* value below 0.05 was considered statistically significant. All statistical analyses were operated by SPSS 17.0 for Windows (SPSS Inc., Chicago, IL, USA).

## 3. Results

The distributions of all genotypes were in accordance with the Hardy–Weinberg Equilibrium in control groups.

### 3.1. NLRP1 Polymorphism

The distributions of NLRP1 (rs8079034 (C>T) and rs878329 (G>C)) genotypes and alleles in the partial seizures patients and the controls are presented in Table 2. The male and female distributions of rs8079034 (C>T) and rs878329 (G>C) genotypes and alleles in the patients and the controls are shown in Tables 3 and 4, respectively. There was no statistically significant difference between patients and controls for the genotype and allele distributions of rs8079034 (C>T) polymorphisms, including all samples, males and females. Between the partial seizures patients and the healthy controls, there was a significant difference in the distribution of the genotypes rs878329 (G>C) (*p* = 0.009). The frequency of genotype with C (CG + CC) was significantly lower among the patients with partial seizures patients relative to the controls (OR = 2.033, 95% CI = 1.290–3.204; *p* = 0.002 for GC + CC versus GG). Moreover, the C allele showed a significant association with partial seizures patients group (OR = 1.801, 95% CI = 1.217–2.667, *p* = 0.003). Interestingly, the statistically significant difference of rs878329 (G>C) genotype and allele frequency only existed among males (OR = 2.492, 95% CI = 1.266–4.906, *p* = 0.008 for GC versus GG; OR = 2.542, 95% CI = 1.344–4.810, *p* = 0.004 for GC + CC versus GG; OR = 2.216, 95% CI = 1.286–3.818, *p* = 0.004 for C versus G), while there was no statistically significant difference among females.

### 3.2. NLRP3 Polymorphism

We analyzed the allele and genotype frequencies of the rs4612666 (C>T), rs10754558 (C>G) and rs2027432 (C>T) for NLRP3 single-nucleotide polymorphisms between partial seizures patients and controls. There was no significant difference in the above mentioned genotypic distribution (Table 5). There was also no significant difference between different genders (data no shown).

### 3.3. P2X7R Polymorphism

The frequencies of the P2X7R of rs3751143 (T>G) and rs208294 (A>G) genotype in the case groups and the controls were investigated and we did not find differences in genotypic distribution or allele distribution (Table 6). There was also no significant difference between males or females (data no shown).

## 4. Discussion

Epilepsy is a chronic neurological disorder, characterized by electrical disturbances in the brain. The important roles of inflammatory processes in relation to modulatory effects of neurotoxic neurotransmitters discharged during excitation or inflammation in the central nervous system have been proved over the last decade, which showed relation to epilepsy or seizures. Large numbers of inflammatory mediators, especially the release of proinflammatory cytokines IL-1*β*, are considered essential in the initiation and regulation of inflammation. Inflammasomes, one type of multiprotein complexes, have been proposed to play key roles in activation of caspase-1 from its inactive proprotein, which then promote the maturation of inflammatory cytokines IL-1*β*.

Inflammasome consists of proinflammatory caspase(s), NLRP family members containing pyrin domain, and an adaptor protein that facilitates the interaction between them. NLRP1 and NLRP3 are the two mostly studied members of inflammasomes and are critical in immune responses. Based on these findings, we explored the relevance between NLRP1 (rs8079034, rs878329) and NLRP3 (rs4612666, rs10754558, and rs2027432) gene polymorphisms and partial seizures in a Chinese Han population for the first time. It was found that there was a significant correlation between NLRP1 (rs878329, G>C) polymorphism and partial seizures in a Chinese Han population. It is consistent with the results of the study carried out by Tan et al., which proved NLRP1 inflammasome was activated in patients with mesial temporal lobe epilepsy [14]. Likewise, in one study about rheumatoid arthritis in Han Chinese, Sui et al. [20] found that the NLRP1 (rs878329, G>C) polymorphism was a risk factor for rheumatoid arthritis. Another study performed by Ekman et al. [21] showed that NLRP1 (rs878329, G>C) polymorphism correlated with psoriasis susceptibility. These results showed that rs878329 (G>C) might be a vital gene in the function of NLRP1.

However, we did not found any significant difference of the rs4612666 (C>T), rs10754558 (C>G) and rs2027432 (C>T) for NLRP3 single-nucleotide polymorphisms between partial seizures patients and controls. The difference between the function of NLRP1 and NLRP3 polymorphism on the epilepsy susceptibility may result from that their cell-specific expression and divergent biological functions; that is, NLRP1 mainly exists in neurons and is essential for the pyroptotic cell death in epilepsy pathogenesis, whereas the NLRP3 is mainly expressed in microglia and is responsive to immune activation. The exact mechanism of the induced activation of the NLRP1 and NLRP3 inflammasomes is unclear. One possible mechanism is that the potassium efflux induces the inflammasome formation. P2X7R is one of important purinergic receptors, promoting the potassium efflux activated by the excessive ATP release. Studies have proved that the P2X7R plays a pivotal role in central nervous system function and may contribute to the progression of several neuropsychiatric disorders, including epilepsy. Based on these clues, we also investigated the relationship between P2X7R (rs3751143, T>G and rs208294, A>G) polymorphisms and partial seizures. Unfortunately, there were no significant differences for the genotypic distribution or allele distribution of rs3751143 (T>G) and rs208294 (A>G) genotypes of P2X7R in the case groups and the controls. These results may be explained by the limitation of this study: the sample size in each genotype is too small to get sufficient statistical power for detecting the slight effect.

The epidemiology of epilepsy in humans indicates that the gender-specific incidence was slightly higher for males than for females which can be explained by the fact that males are more liable to be exposed to the higher risk factors for epilepsy such as central nervous system infection, head injury, and stroke [22]. Accordingly, one of the surprising results of this research is that the genetic variation of rs878329 (G>C) seems to contribute to the susceptibility of partial seizures, while no difference was observed between female patients and controls. Additionally, sex hormones, such as androgen, estrogen, and progesterone, along with their metabolites, play a role in brain network formation and can affect the activity of inflammasomes and neuroimmune system. Therefore, the gender difference found in the present study can be at least partially attributed to the interaction between steroid hormones and inflammasomes in neuroinflammatory process [23–25].

## 5. Conclusion

In summary, to our knowledge, this is the first report exploring the potential relevance on molecular mechanism between NLPR1, NLRP3, and P2X7R polymorphisms and partial seizures in a Chinese Han population, and our data suggest that there was a significant correlation between NLRP1 (rs878329, G>C) polymorphism and partial seizures in the study population. Although our data suggest a potential role of NLPR1 polymorphisms in susceptibility to seizures, these results need to be consolidated by further studies involving other ethnics and a larger group of seizures patients.

## Figures and Tables

**Table 1:** Primers of target genes used in the PCR.

SNP	Ancestor allele	Primer sequence	Product size
NLRP1	C	5′-TGATGGTCTGATTCATGCCC-3′ (forward)	98 bp
(rs8079034)		5′-GTAGTTGCTAGGCAATGCGG-3′ (reverse)	

NLRP1	G	5′-ATCCACTCAACTCCCTCAAC-3′ (forward)	111 bp
(rs878329)		5′-CAACATGAGACCAGTCCTTG-3′ (reverse)	

NLRP3	C	5′-TTCCTTTTCCATTTGGTGGA-3′ (forward)	200 bp
(rs4612666)		5′-AGATGGTGGTGGTGATGGTT-3′ (reverse)	

NLRP3	C	5′-GGTCACCAAGAGGAACATCC-3′ (forward)	160 bp
(rs10754558)		5′-GGTGGAGTGTCGGAGAAGAG-3′ (reverse)	

NLRP3	C	5′-TGAGGCCTTTAAAACAGAGC-3′ (forward)	116 bp
(rs2027432)		5′-GAGCATTCTCTCTGCAGTTC-3′ (reverse)	

P2RX7	T	5′-TTCCTGGACAACCAGAGGAG-3′ (forward)	240 bp
(rs3751143)		5′-TCCTGGTAGAGCAGGAGGAA-3′ (reverse)	

P2RX7	A	5′-GTTAGGATGGGCTTGATGGA-3′ (forward)	227 bp
(rs208294)		5′-CACCAGGCAGAGACTTCACA-3′ (reverse)	

**Table 2:** Genotypic and allelic distribution of the NLRP1 gene between all patients (*n* = 163) and controls (*n* = 201).

SNP	Genotype/allele	Case (%)	Control (%)	*p* value^a^(*χ*^2^)	OR (95% CI)	*p* value^b^
rs8079034	CC	120 (73.6)	153 (76.1)	0.752 (0.569)	1.00	Referent
	CT	37 (22.7)	43 (21.4)		0.911 (0.553–1.503)	0.717
	TT	6 (3.7)	5 (2.5)		0.654 (0.195–2.193)	0.491
	CT + TT	43 (26.4)	48 (23.9)	0.584 (0.300)	0.876 (0.544–1.409)	0.584
	C	277 (85.0)	349 (86.8)	0.475 (0.509)	1.00	Referent
	T	49 (15.0)	53 (13.2)		0.858 (0.565–1.306)	0.476

rs878329	GG	123 (75.5)	121 (60.2)	0.009^*∗*^ (9.486)	1.00	Referent
	GC	35 (21.5)	70 (34.8)		2.033 (1.262–3.276)	0.004^*∗*^
	CC	5 (3.0)	10 (5.0)		2.033 (0.675–6.123)	0.207
	GC + CC	40 (24.5)	80 (39.8)	0.002^*∗*^ (9.486)	2.033 (1.290–3.204)	0.002^*∗*^
	G	281 (86.2)	312 (77.6)	0.003^*∗*^ (8.782)	1.00	Referent
	C	45 (13.8)	90 (22.4)		1.801 (1.217–2.667)	0.003^*∗*^

CI, confidence interval; OR, odds ratio.

^a^ *p* value for genotype and allele frequencies in cases and controls using 2-sided *χ*^2^ test.

^b^ *p* values adjusted by age and gender using logistic regression.

^*∗*^ *p* < 0.05.

**Table 3:** Genotypic and allelic distribution of the NLRP1 gene between male patients (*n* = 89) and controls (*n* = 99).

SNP	Genotype/allele	Case (%)	Control (%)	*p* value^a^(*χ*^2^)	OR (95% CI)	*p* value^b^
rs8079034	CC	63 (70.8)	78 (78.8)	0.432 (1.678)	1.00	Referent
	CT	23 (25.8)	18 (18.2)		0.632 (0.314–1.274)	0.199
	TT	3 (3.4)	3 (3.0)		0.808 (0.158–4.140)	0.798
	CT + TT	26 (29.2)	21 (21.2)	0.206 (1.600)	0.652 (0.336–1.267)	0.207
	C	149 (83.7)	174 (87.9)	0.246 (1.347)	1.00	Referent
	T	29 (16.3)	24 (12.1)		0.709 (0.395–1.270)	0.247

rs878329	GG	69 (77.5)	57 (57.6)	0.015^*∗*^ (8.466)	1.00	Referent
	GC	17 (19.1)	35 (35.4)		2.492 (1.266–4.906)	0.008^*∗*^
	CC	3 (3.4)	7 (7.0)		2.825 (0.698–11.423)	0.145
	GC + CC	20 (22.5)	42 (42.4)	0.004^*∗*^ (8.441)	2.542 (1.344–4.810)	0.004^*∗*^
	G	155 (87.1)	149 (75.3)	0.004^*∗*^ (8.467)	1.00	Referent
	C	23 (12.9)	49 (24.7)		2.216 (1.286–3.818)	0.004^*∗*^

CI, confidence interval; OR, odds ratio.

^a^ *p* value for genotype and allele frequencies in cases and controls using 2-sided *χ*^2^ test.

^b^ *p* values adjusted by age and gender using logistic regression.

^*∗*^ *p* < 0.05.

**Table 4:** Genotypic and allelic distribution of the NLRP1 gene between female patients (*n* = 74) and controls (*n* = 102).

SNP	Genotype/allele	Case (%)	Control (%)	*p* value^a^(*χ*^2^)	OR (95% CI)	*p* value^b^
rs8079034	CC	57 (77.0)	75 (73.5)	0.513 (1.336)	1.00	Referent
	CT	14 (18.9)	25 (24.5)		1.357 (0.648–2.843)	0.418
	TT	3 (4.1)	2 (2.0)		0.507 (0.082–3.133)	0.465
	CT + TT	17 (23.0)	27 (26.5)	0.597 (0.280)	1.207 (0.601–2.425)	0.597
	C	128 (86.5)	175 (86.8)	0.851 (0.035)	1.00	Referent
	T	20 (13.5)	29 (14.2)		1.061 (0.574–1.959)	0.851

rs878329	GG	54 (73.0)	64 (62.7)	0.350 (2.009)	1.00	Referent
	GC	18 (24.3)	35 (34.3)		4.641 (0.836–3.219)	0.150
	CC	2 (2.7)	3 (3.0)		1.266 (0.204–7.854)	0.800
	GC + CC	20 (27.0)	38 (37.3)	0.154 (2.031)	1.603 (0.836–3.075)	1.603
	G	126 (85.1)	163 (79.9)	0.206 (1.599)	1.00	Referent
	C	22 (14.9)	41 (20.1)		1.441 (0.817–2.542)	0.208

CI, confidence interval; OR, odds ratio.

^a^ *p* value for genotype and allele frequencies in cases and controls using 2-sided *χ*^2^ test.

^b^ *p* values adjusted by age and gender using logistic regression.

**Table 5:** Genotypic and allelic distribution of the NLRP3 gene between all patients (*n* = 163) and controls (*n* = 201).

SNP	Genotype/allele	Case (%)	Control (%)	*p* value^a^(*χ*^2^)	OR (95% CI)	*p* value^b^
rs4612666	CC	44 (27.0)	59 (29.4)	0.220 (3.026)	1.00	Referent
	CT	90 (55.2)	94 (46.8)		0.779 (0.479–1.266)	0.313
	TT	29 (17.8)	48 (23.9)		1.234 (0.675–2.258)	0.495
	CT + TT	119 (73.0)	142 (70.6)	0.584 (0.300)	0.890 (0.562–1.410)	0.619
	C	178 (54.6)	212 (52.7)	0.616 (0.252)	1.00	Referent
	T	148 (45.4)	190 (47.3)		1.078 (0.804–1.445)	0.616

rs10754558	CC	50 (30.7)	76 (37.8)	0.313 (2.322)	1.00	Referent
	CG	84 (51.5)	89 (44.3)		0.697 (0.438–1.110)	0.128
	GG	29 (17.8)	36 (17.9)		0.817 (0.446–1.496)	0.512
	CG + GG	113 (69.3)	125 (62.2)	0.155 (2.025)	0.728 (0.470–1.128)	0.155
	C	184 (56.4)	241 (60.0)	0.340 (0.912)	1.00	Referent
	G	142 (43.6)	161 (40.0)		0.866 (0.644–1.164)	0.340

rs2027432	CC	151 (92.6)	180 (89.5)	0.549 (1.200)	1.00	Referent
	CT	11 (6.8)	20 (10.0)		1.525 (0.708–3.284)	0.281
	TT	1 (0.6)	1 (0.5)		0.839 (0.052–13.525)	0.901
	CT + TT	12 (7.4)	21 (10.5)	0.308 (1.040)	1.468 (0.699–3.082)	0.310
	C	313 (96.0)	380 (94.5)	0.352 (0.867)	1.00	Referent
	T	13 (4.0)	22 (5.5)		1.394 (0.691–2.812)	0.354

CI, confidence interval; OR, odds ratio.

^a^ *p* value for genotype and allele frequencies in cases and controls using 2-sided *χ*^2^ test.

^b^ *p* values adjusted by age and gender using logistic regression.

**Table 6:** Genotypic and allelic distribution of the P2RX7 gene between all patients (*n* = 163) and controls (*n* = 201).

SNP	Genotype/allele	Case (%)	Control (%)	*p* value^a^(*χ*^2^)	OR (95% CI)	*p* value^b^
rs3751143	TT	94 (57.7)	127 (63.2)	0.451 (1.592)	1.00	Referent
	TG	59 (36.2)	66 (32.8)		0.828 (0.533–1.287)	0.401
	GG	10 (6.1)	8 (4.0)		0.592 (0.225–1.558)	0.288
	GT + GG	69 (42.3)	74 (36.8)	0.284 (1.148)	0.794 (0.520–1.211)	0.284
	T	247 (75.8)	320 (79.6)	0.215 (1.537)	1.00	Referent
	G	79 (24.2)	82 (20.4)		0.801 (0.564–1.138)	0.215

rs208294	AA	58 (35.6)	69 (34.3)	0.741 (0.599)	1.00	Referent
	AG	83 (50.9)	99 (49.3)		1.003 (0.636–1.580)	0.991
	GG	22 (13.5)	33 (16.4)		1.261 (0.663–2.397)	0.480
	AG + GG	105 (64.4)	132 (65.7)	0.830 (0.062)	1.057 (0.685–1.630)	0.803
	A	199 (61.0)	237 (59.0)	0.568 (0.327)	1.00	Referent
	G	127 (39.0)	165 (41.0)		1.091 (0.809–1.470)	0.568

CI, confidence interval; OR, odds ratio.

^a^ *p* value for genotype and allele frequencies in cases and controls using 2-sided *χ*^2^ test.

^b^ *p* values adjusted by age and gender using logistic regression.
